# A Manual of Hygiene and Sanitation

**Published:** 1910-11

**Authors:** 


**A Manual of Hygiene and Sanitation.** By **Seneca Egbert**, M.D., Dean and Professor of Hygiene in the Medico-Chirurgical College, Philadelphia. Fifth edition. 12 mo, 508 pages. With 97 illustrations. Philadelphia and New York: Lea & Febiger. 1910. (Cloth, $2.25, net.)

This is the fifth edition of Professor Egbert's wellknown and popular manual on hygiene. The new revision is a little larger and better than its predecessors in that it contains some new material and is brought well up to date. The author has quoted very fully from the "Report on Human Vitality—Its Waste and Conservatism" by Professor Irving Fisher. J. A. R.

